# Machine learning-based Radiomics analysis for differentiation degree and lymphatic node metastasis of extrahepatic cholangiocarcinoma

**DOI:** 10.1186/s12885-021-08947-6

**Published:** 2021-11-24

**Authors:** Yong Tang, Chun Mei Yang, Song Su, Wei Jia Wang, Li Ping Fan, Jian Shu

**Affiliations:** 1 School of Computer Science and Engineering, University of Electronic Science and Technology of China, No. 4, Section 2, North Jianshe Road, Chengdu, 610054 Sichuan China; 2 Department of Radiology, The Affiliated Hospital of Southwest Medical University, and Nuclear Medicine and Molecular Imaging Key Laboratory of Sichuan Province, Luzhou, 646000 Sichuan China; 3 Department of Hepatobiliary Surgery, The Affiliated Hospital of Southwest Medical University, 25 Taiping Street, Luzhou, 646000 Sichuan China; 4 School of Information and Software Engineering, University of Electronic Science and Technology of China, No. 4, Section 2, North Jianshe Road, Chengdu, 610054 Sichuan China; 5 Department of Ultrasound, The Affiliated Hospital of Southwest Medical University, 25 Taiping Street, Luzhou, 646000 Sichuan China

**Keywords:** Extrahepatic cholangiocarcinoma, Cell differentiation, Lymphatic metastasis, Machine learning, Radiomics

## Abstract

**Background:**

Radiomics may provide more objective and accurate predictions for extrahepatic cholangiocarcinoma (ECC). In this study, we developed radiomics models based on magnetic resonance imaging (MRI) and machine learning to preoperatively predict differentiation degree (DD) and lymph node metastasis (LNM) of ECC.

**Methods:**

A group of 100 patients diagnosed with ECC was included. The ECC status of all patients was confirmed by pathology. A total of 1200 radiomics features were extracted from axial T1 weighted imaging (T1WI), T2-weighted imaging (T2WI), diffusion weighted imaging (DWI), and apparent diffusion coefficient (ADC) images. A systematical framework considering combinations of five feature selection methods and ten machine learning classification algorithms (classifiers) was developed and investigated. The predictive capabilities for DD and LNM were evaluated in terms of area under precision recall curve (AUPRC), area under the receiver operating characteristic (ROC) curve (AUC), negative predictive value (NPV), accuracy (ACC), sensitivity, and specificity. The prediction performance among models was statistically compared using DeLong test.

**Results:**

For DD prediction, the feature selection method joint mutual information (JMI) and Bagging Classifier achieved the best performance (AUPRC = 0.65, AUC = 0.90 (95% CI 0.75–1.00), ACC = 0.85 (95% CI 0.69–1.00), sensitivity = 0.75 (95% CI 0.30–0.95), and specificity = 0.88 (95% CI 0.64–0.97)), and the radiomics signature was composed of 5 selected features. For LNM prediction, the feature selection method minimum redundancy maximum relevance and classifier eXtreme Gradient Boosting achieved the best performance (AUPRC = 0.95, AUC = 0.98 (95% CI 0.94–1.00), ACC = 0.90 (95% CI 0.77–1.00), sensitivity = 0.75 (95% CI 0.30–0.95), and specificity = 0.94 (95% CI 0.72–0.99)), and the radiomics signature was composed of 30 selected features. However, these two chosen models were not significantly different to other models of higher AUC values in DeLong test, though they were significantly different to most of all models.

**Conclusion:**

MRI radiomics analysis based on machine learning demonstrated good predictive accuracies for DD and LNM of ECC. This shed new light on the noninvasive diagnosis of ECC.

## Background

Cholangiocarcinoma (CCA), categorized as intrahepatic (ICC) and extrahepatic (ECC) forms, is a malignant neoplasm arising from the biliary epithelium, representing an estimated 3% of all gastrointestinal system malignancies [1]. ECC, originating from the bile ducts outside the liver parenchyma, accounts for approximately 80% of all CCA. ECC is divided into two types of perihilar and distal cholangiocarcinoma [1, 2]. It was reported that the incidence and mortality rates of ECC have been increasing gradually worldwide over the last decades, although it’s not as established as ICC [3]. The prognosis of ECC and ICC still remains poor. The only effective way to cure ECC is complete surgical resection. However, it is only appropriate for patients with well-localized lesions [4]. The curative rate of ECC has been low for patients in advanced stages. Even with complete resection of the tumors, most patients may encounter a poor prognosis (e.g., local recurrence, distant metastasis, or death), which is associated with the differentiation degree (DD) and lymph node metastasis (LNM) [5, 6]. Therefore, it is crucial to accurately evaluate ECC, especially DD and LNM of the tumor, in order to select optimal treatment strategies and determine prognosis.

Several imaging techniques could diagnose ECC, including ultrasonography (US) [7], computerized tomography (CT) [8], positron emission computerized tomography (PET-CT) [9], magnetic resonance imaging (MRI) [10, 11], and cholangioscopy [12], etc. At present, MRI has become the imaging modality of choice for diagnosis and staging of CCA, with high soft-tissue contrast to help better detect and identify the infiltrating tumors [10]. It has been reported that MRI techniques are helpful in displaying the stricture morphology of bile ducts clearly, such as irregularity and wall thickness [11], regional lymph node, distant metastases, and survival outcomes of CCA [13]. This imaging method could provide an accurate preoperative evaluation of CCA, result in better treatment selection and improved prognosis [13]. However, conventional techniques still have some disadvantages. They rely on subjective visual evaluations of the radiologists to a large extent and are not quantitative methods for predicting DD and LNM of ECC [14]. It is hard to analyze the large number of digital features embedded in the images involving cells, physiology, and genetic variation of patients, which cannot be recognized by human eyes [14].

Radiomics, a recently introduced methodology, describes quantitative computerized algorithm-based feature extraction from traditional imaging data, including CT, MRI, or PET images [15–17]. This technique, opposing to the subjective visual interpretation of the radiologists, quantifies the heterogeneity of lesions objectively. Previous studies have suggested improvements in the preoperative prediction of LNM by using radiomics-based approaches to lung adenocarcinoma, gastric cancer, colorectal cancer, bladder cancer, and breast cancer [18–22]. In addition, radiomics has been applied to predict the histopathological grades of tumors such as soft tissue sarcomas and gliomas [23, 24].

Recently, it has been reported to use radiomics to diagnose and evaluate CCA. Some protein expressions of CCA and early recurrence of ICC could be predicted based on radiomics methods [25–27]. To our knowledge, radiomics models have been used in predicting LNM of biliary tract cancers or ICC and determining its prognostic value [28–30]. Therefore, the aim of this study is to develop machine learning-based radiomics models to predict DD and LNM of ECC.

## Methods

**Fig. 1:**
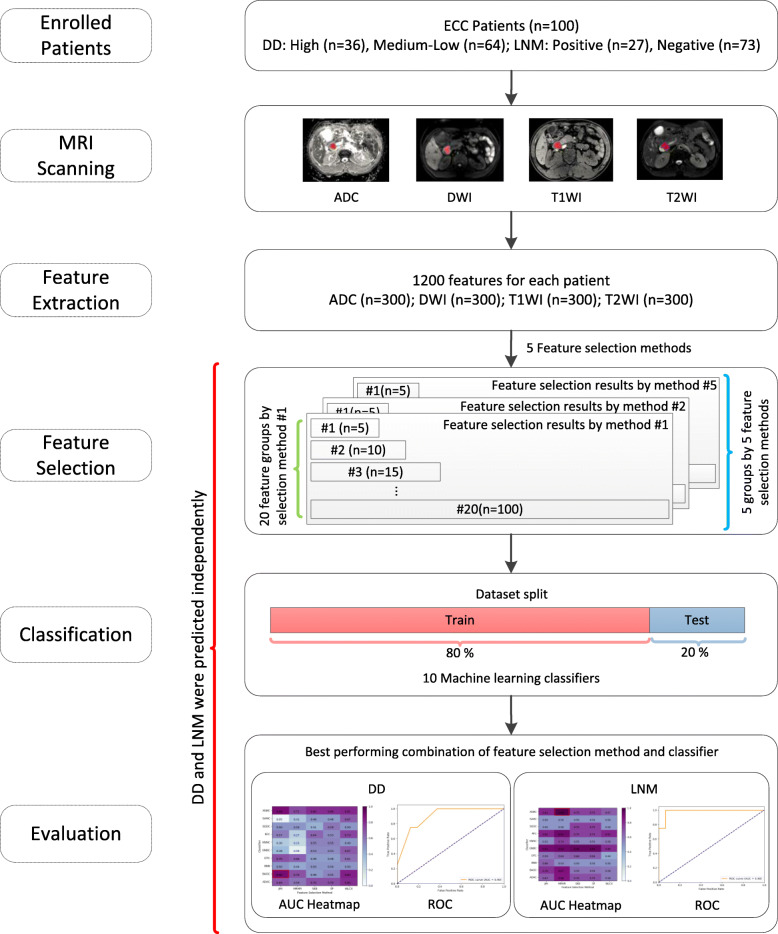
Radiomics development flowchart of this study

The flowchart of this study was illustrated in Fig. 1. We first retrospectively collected patient records and obtained the MRI scanning. The texture features of tumors were extracted from the MRI images. For the independent predictions of DD and LNM, the machine learning-based radiomics were developed in two steps, including feature selection and classification, before final evaluations. The predictions of DD and LNM were conducted separately. Namely, the two steps were repeated for DD and LNM, respectively.

### Patients characteristics

Ethical approval for this retrospective study was obtained from the Ethical Committee of the Affiliated Hospital of Southwest Medical University (KY2019063). The procedures of this study strictly followed the standard rules and regulations of the hospital. The patient informed consent was waived, and all patient identification information was removed. The inclusion criteria were (1) all patients who experienced MRI examinations no more than two weeks before surgical resection, and (2) all patients who underwent surgical excisions and pathological examinations. The exclusion criteria were (1) patients whose lesions were not identified, (2) patients whose MRI examinations sequences were incomplete, and (3) patients whose MRI images in which the lesions were too small to be identified. Initially, we collected 144 patients based on clinical data. However, 39 patients were excluded because of obscure MRI images or incomplete sequences, and five patients were excluded because of existing inconspicuous lesions without being identified. As a result, a group of ECC patients (*n* = 100) diagnosed and treated in the hospital between January 2011 to December 2018 were included in our study. Due to the low incidence rate of ECC, the sample size in the present study was limited. More samples could be included in future studies.

Clinical data (e.g., gender, age, primary tumor site, and lesion size) and the baseline appearance of MRI were obtained from medical records. The ECC status ascertainment (pathological grades and lymphatic status) was confirmed by pathology reports and reviewed by an abdominal pathological expert with ten years of experience.

### MRI acquisition

All patients underwent preoperational MRI scans using a 3.0 T MRI scanner (Achieva 3.0 T, Philips, Amsterdam, Netherlands) with a 16-channel abdominal coil. The scanning range extended from the top of the diaphragm to the lower edge of the liver. For each patient, the following MRI sequences were obtained: an axial T1 high-resolution isotropic volume excitation (THRIVE) sequence (T1 weighted imaging, T1WI) (TR = 3.1 ms, TE = 1.44 ms, flip angle = 10°, matrix = 244 × 186, a field of view (FOV) = 280 mm × 305 mm, number of excitations (NEX) =1, section thickness = 3 mm, gap = − 1.5 mm), an axial fat-suppressed turbo spin echo (TSE) T2-weighted imaging sequence (T2WI) (TR = 1610 ms, TE = 70 ms, flip angle = 90°, matrix = 176 × 201, FOV = 280 mm × 305 mm, NEX = 2, section thickness = 7 mm, gap = 1 mm), an axial diffusion weighted imaging (DWI) (TR = 934 ms, TE = 52 ms, flip angle = 90°, matrix = 100 × 124, FOV = 280 mm × 305 mm, NEX = 4, section thickness = 7 mm, gap = 1 mm, b values = 0,600 and 800 s/mm^2^), a coronal TSE T2WI sequence, an axial dual-echo T1WI breath-hold gradient-echo sequence for the acquisition of in-phase and out-of-phase images, MR cholangiopancreatography (MRCP), and a T1-weighted dynamic contrast-enhanced MRI. ADC images were obtained by reconstructing DWI images in the post-processing workstation (Philips Extended MR Workspace 2.6.3.4). Finally, the T1WI, T2WI, DWI (b = 800 s/mm^2^), and ADC images were used in this study.

### Radiomics features extraction

**Fig. 2:**
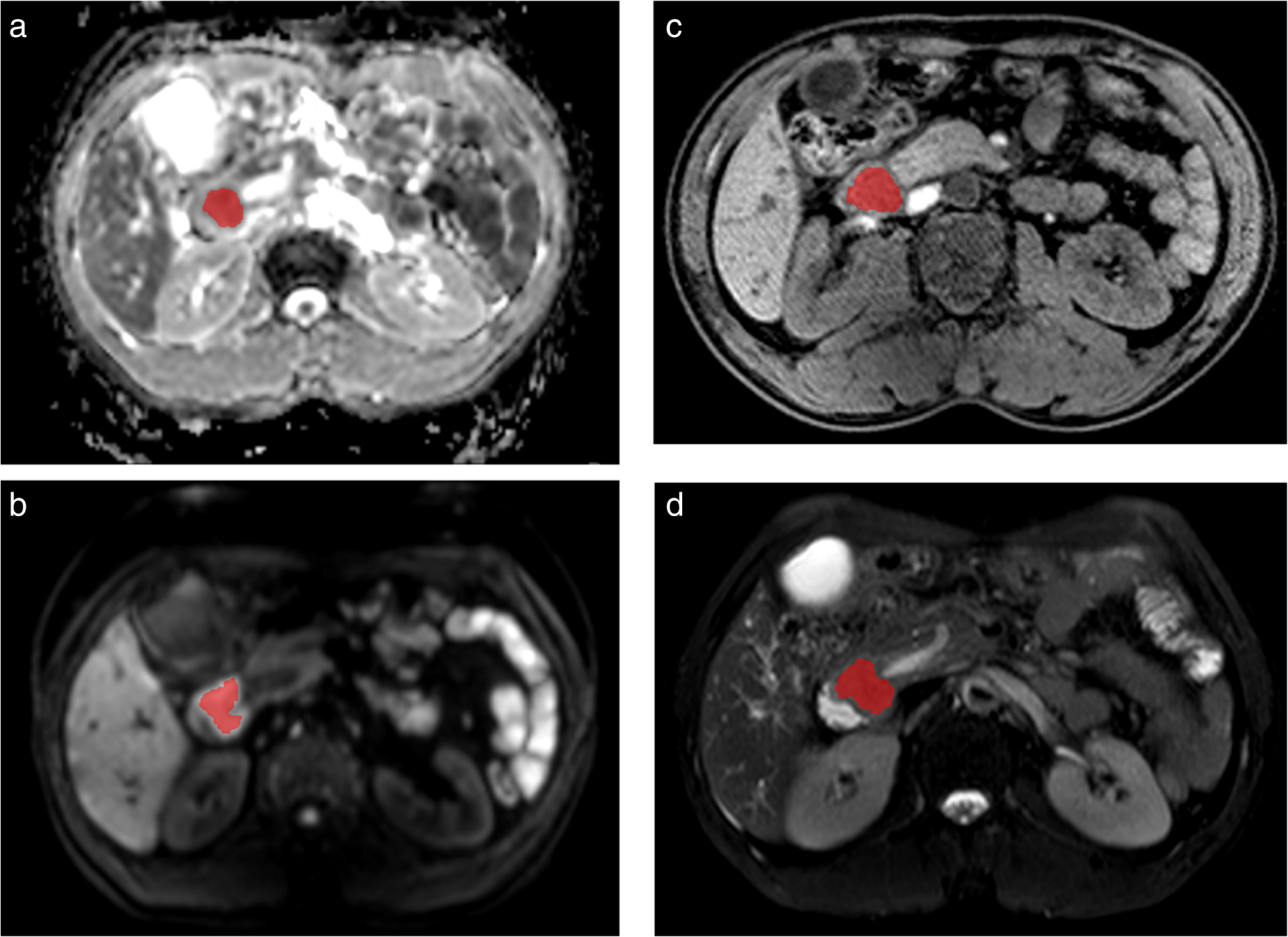
ROI was placed on the maximum section of the tumor, avoiding adjacent vessels and bile duct on ADC (a), DWI (b), T1WI (c), and T2WI (d), respectively

All MRI images were extracted from the same machine using the same scanning parameters in this study. Therefore, no data preprocess was executed. All images were examined by an expert radiologist with eight years of experience in abdominal radiological diagnosis. Using MaZda software (version 4.6),^1^ the regions of interest (ROI) of the lesion on the maximum section of the tumor were delineated manually, avoiding adjacent vessels and bile duct, as shown in Fig. 2.

Four MRI sequence features were extracted from MRI images, including T1WI, T2WI, DWI (b = 800 s/mm^2^), and ADC images. Six common feature groups, including histogram, absolute gradient, gray-level co-occurrence matrix, run-length matrix, autoregressive model, wavelet transform, were extracted using MaZda. Each sequence had 300 features. As a result, a total of 1200 features were extracted from the four MRI sequences for each patient.

### Feature selection

First, we applied feature selection methods to reduce feature dimensions before conducting classification predictions. In this study, five feature selection methods, including joint mutual information (JMI) [31], minimum redundancy maximum relevance (MRMR) [32], select K best-using analysis of variance (SKB), select percentile (SP) [33], and Wilcoxon (WLCX) [34], were applied to the 1200 features to select the most significant features for DD and LNM, respectively. These filter-based methods were frequently applied in studies [35]. The features were ranked using the above feature selection methods based on joint mutual information (JMI) [35–37], redundancy and relevance (MRMR) [37–39], ANOVA F-value (SKB, SP) [37, 38], *p*-value (WLCX) [35, 37, 39], respectively. For each feature selection method, different number of selected features (*n* = 5, 10, 15, ..., 100) were selected for further classifications. In other words, each feature selection method generated 20 groups of selected features in different numbers ranged from n = 5 to *n* = 100 with an increment of five. This approach allowed sufficient searches of significant features. As a result, we obtained 100 groups (*n* = 20 × 5) of selected features (20 for each of the five feature selection methods) for DD and LNM, respectively. These selected feature groups would be later used to conduct independent classifications using different machine learning classifiers.

### Classification prediction

After feature selection, we applied machine learning classifiers to the selected features. In this study, the predictions of DD (high, medium-low) and LNM (positive, negative) were two separated binary classifications and conducted independently. For all of the 100 groups of selected features obtained by the five feature selection methods, ten machine learning classifiers (Table 1) were applied to evaluate the final classification performance of the different combinations of feature selection methods and classifiers. Thus, we conducted systematical evaluations of 1000 cases (*n* = 5 × 20 × 10). To evaluate the performance of classifiers, the metrics including area under the receiver operating characteristic (ROC) curve (AUC) [40], accuracy (ACC), sensitivity, and specificity were calculated using the test set for DD and LNM, respectively. Using AUC as the major metric, we organized these results into 20 groups according to the number of selected features (n = 5, 10, 15, ..., 100). Results of all groups were later organized and illustrated as heatmaps, from which we further identified and reported the highest AUC value and the corresponding heatmap. It’s worth noting that multiple AUC values achieved by multiple methods should be compared statistically using DeLong test [41]. If a model has a better AUC value and at the same time is significant in DeLong test in comparing with other models, we can acclaim that this model is optimal and significantly different to other models. While, though a model has better AUC value but is not significant in DeLong test in comparing with other models, we should avoid overstating that this model is significantly superior compared to other models, since the model is not significant in DeLong test in comparing with other models. Therefore, in reporting and comparing the performance of models, the DeLong tests should be reported no matter the test results were significant or not [42–49]. In line with previous radiomics studies involving comparing performance of multiple models [43, 44, 46–48], we conducted DeLong test [41] to evaluate the statistical differences between models. Namely, pairwise DeLong tests were performed for models in classifications of DD and LNM, respectively. Statistically, for any given two models, a significant DeLong test result (*p*-value < 0.05) indicates the two models are significantly different. All methods used in the feature selection and classification were implemented in Python (version 3.6.3) using the publicly available Pandas library (version 0.24.2), NumPy library (version 1.15.1), SciPy library (version 1.0.0), and Scikit-learn library (version 0.19.1). DeLong test was implemented and performed in Python according to the algorithm of the original paper [41]. We further provided the source codes we developed in this study for interested researchers at GitHub (https://github.com/gracewang723/EC-paper).

**Table 1:** Classification machine learning algorithms

Number	Abbreviation	Algorithm
1	ADAC	Ada Boosting Classifier
2	BAGC	Bagging Classifier
3	BNB	Bernoulli Naïve Bayesian
4	DTC	Decision Tree Classifier
5	GNBC	Gaussian Naïve Bayesian Classifier
6	KNNC	K Nearest Neighborhood Classifier
7	RFC	Random Forest Classifier
8	SGDC	Stochastic Gradient Descent Classifier
9	SVMC	Support Vector Machine Classifier
10	XGBC	eXtreme Gradient Boosting Classifier

### Statistical analysis

The age and the lesion size were expressed as mean ± standard deviation (SD) when the distribution of data was normal or as median when it was outside the bounds of normality. The variables were compared using independent t-tests or Wilcoxon Rank Sum tests, when appropriate. Gender was compared using the chi-squared test. The above statistical analyses were conducted using SPSS 25. A two-sided *p* value < 0.05 was considered significant. The classification performance was assessed using the ROC curve and AUC. The models were compared with DeLong test [41], and the difference between models was considered statistically significant with *p*-value < 0.05.

## Results

### Patients

Table 2 provided a summary of the patient characteristics of this study (*n* = 100). There were 54 males (54%) and 46 females (46%) with an age range of 28–83 and a median age of 59.5. All tumors were confirmed to be adenocarcinomas and were divided into high (*n* = 36), medium (*n* = 46), and low (*n* = 18) differentiation groups based on the World Health Organization classification of digestive system tumors (4th edition). Given that the sample size is too small in the low differentiation group, patients were classified into high (n = 36) and medium-low differentiation groups (*n* = 64) in our study. Among them, 27 and 73 patients were found to have positive LNM (27%) and negative LNM (73%), respectively. We further randomly divided the patients into two cohorts, namely one training set (*n* = 80, 80%) and one test set (*n* = 20, 20%) to ensure that no data of a given individual appear in both sets in order to avoid bias.

**Table 2:** Patient characteristics

Characteristics	Training cohort			Testing cohort			Training cohort			Testing cohort		
	LNM	Non-LNM	*P*	LNM	Non-LNM	*P*	High	Medium-low	*P*	High	Medium-low	*P*
Sex			0.823			0.180			0.502			0.655
Male	12	29		2	11		21	22		2	9	
Female	11	28		2	5		11	26		2	7	
Age (years)	53.4 ± 11.0	57.7 ± 9.7	0.092	53.8 ± 12.0	60.8 ± 7.7	0.159	56.6 ± 7.7	57.2 ± 11.9	0.814	62.8 ± 9.3	56.0 ± 7.8	0.151
Lesion size (cm)	1.9 ± 0.9	1.3 ± 0.8	0.008	1.6 ± 0.3	1.2 ± 0.5	0.154	1.4 ± 1.0	1.5 ± 7.6	0.443	1.1 ± 0.4	1.4 ± 0.5	0.254

LNM, lymph node metastases

Lesion size was defined as the maximum diameter on transverse images

The values of age and lesion size were expressed as mean ± SD

A two-sided *P* value < 0.05 was considered significant

### Feature selection and Radiomics signature construction

#### DD prediction

Using the extracted 1200 radiomics features, we applied each of the five feature selection methods to obtain 20 groups of selected features of different numbers of features, namely *n* = 5, 10, ..., 100. We further applied ten classification methods to each of the 20 selected feature groups to conduct the classifications for DD prediction. Therefore, for each group, 50 combinations of five feature selection methods and ten classifiers were systematically investigated. Thus, we have evaluated the performance of 1000 = 20*50 possible combinations. The performance metrics were applied to the independent test set (*n* = 20).

**Fig. 3:**
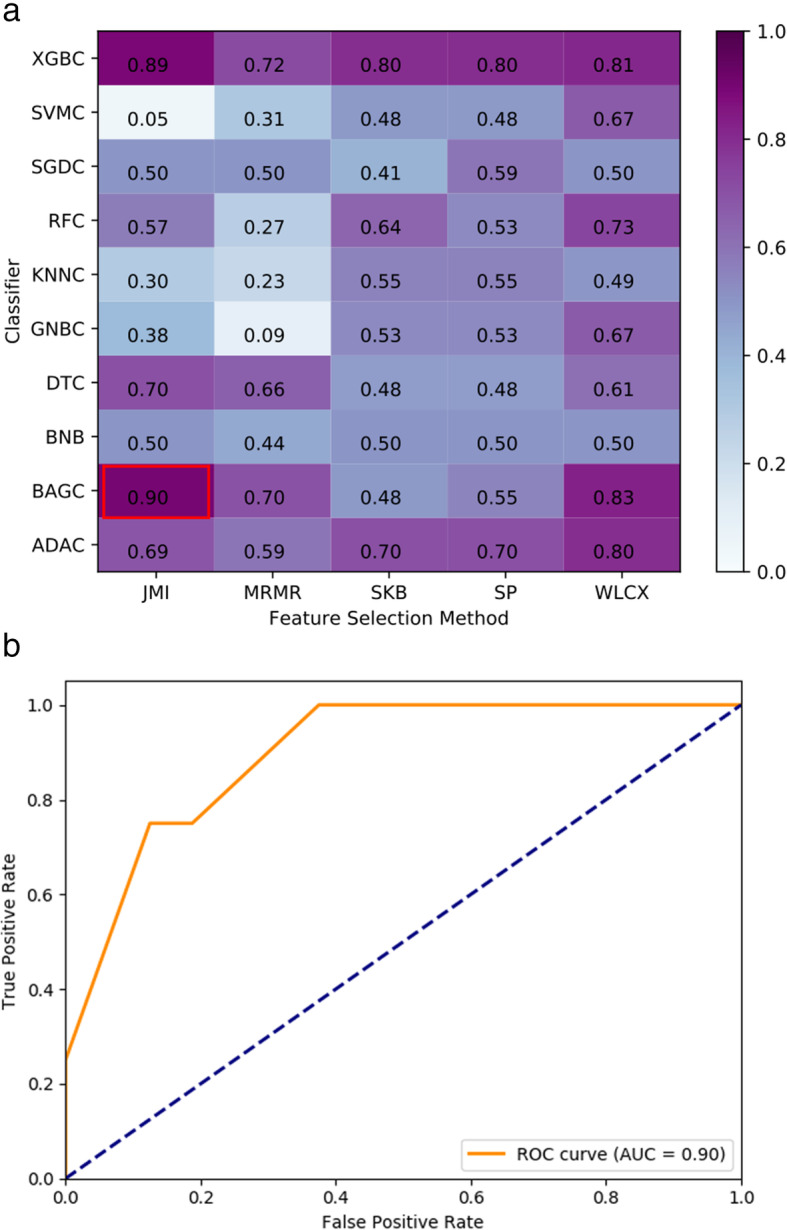
DD prediction AUC heatmap and ROC. (a) Combinations of feature selection methods and classifiers; (b) ROC for the best performing combination of feature selection method JMI and classifier BAGC (feature number *n* = 5, AUC = 0.90)

Among all of the selected groups, the combination of feature selection method JMI and classifier BAGC achieved the highest performance with AUPRC = 0.65, AUC = 0.90 (95% CI 0.75–1.00), ACC = 0.85 (95% CI 0.69–1.00), sensitivity = 0.75 (95% CI 0.30–0.95), and specificity = 0.88 (95% CI 0.64–0.97). As listed in Table 3, the number of selected features in this group was five, including two ADC features, one DWI feature, one T1WI feature, and one T2WI feature. For this group, we plotted the heatmap of AUC values in Fig. 3a and the ROC in Fig. 3b, respectively. The corresponding DD radiomics with the best AUC included five features of two ADC features, one DWI feature, one T1WI feature, and one T2WI feature. In DeLong test, the combination of JMI and BAGC was found significantly different to most of the rest models. However, no statistically difference was observed to other combinations of high AUC values. For example, the highest combination (JMI and BAGC, AUC = 0.90) was not significantly different to the second highest combination (JMI and XGBC, AUC = 0.89) (*p*-value = 0.9004, DeLong test).

**Table 3:** The selected features with the best performance for DD

Number	Sequence	Feature
1	ADC	S(4,0)Contrast
2	ADC	S(5,0)Contrast
3	DWI	S(4,4)DifEntrp
4	T1WI	S(4,4)Entropy
5	T2WI	S(4,4)DifEntrp

Axial T1-precontrast weighted imaging, T1WI; axial T2-weighted imaging, T2WI; axial diffusion weighted imaging, DWI; Apparent diffusion coefficient, ADC

#### LNM prediction

**Fig. 4:**
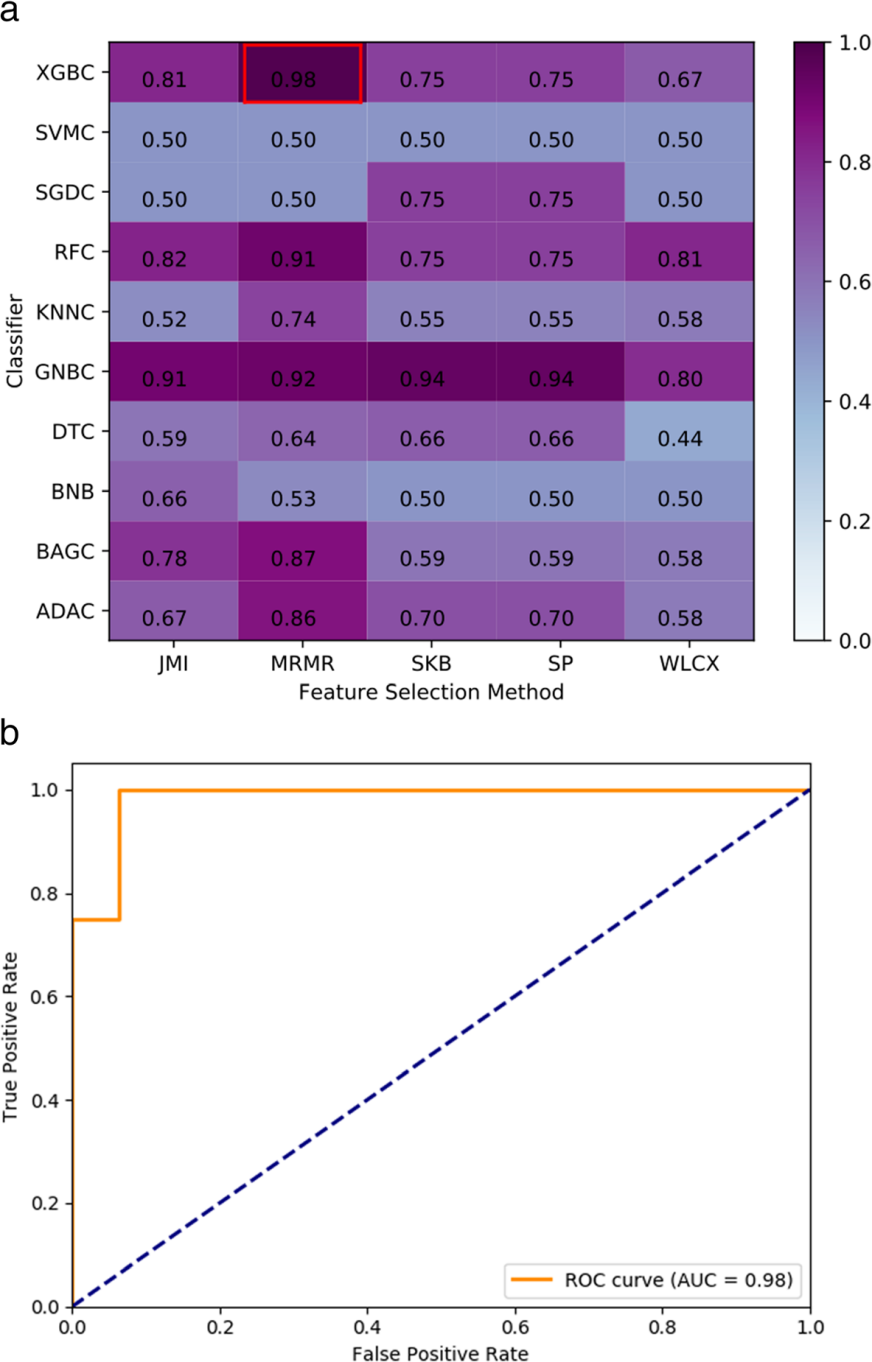
LNM prediction AUC heatmap and ROC. (a) Combinations of feature selection methods and classifiers; (b) ROC for the best performing combination of feature selection method MRMR and classifier XGBC (feature number *n* = 30, AUC = 0.98)

Similarly, the LNM classification was also conducted using combinations of five feature selection methods and ten classifiers based on the independent test set (*n* = 20). As listed in Table 4, the group with 30 selected features, including seven ADC features, seven DWI features, eight T1WI features, and eight T2WI features, achieved the highest performance using feature selection method MRMR and classifier XGBC with AUPRC = 0.95, AUC = 0.98 (95% CI 0.94–1.00), ACC = 0.90 (95% CI 0.77–1.00), sensitivity = 0.75 (95% CI 0.30–0.95), and specificity = 0.94 (95% CI 0.72–0.99). For this LNM prediction, we plotted the heatmap of AUC in Fig. 4a and ROC in Fig. 4b, respectively. Similar to DD, in DeLong test, the combination of MRMR and XGBC was found significantly different to most of the rest models. However, no statistical difference was observed to other combinations of high AUC values. For example, the highest combination (MRMR and XGBC, AUC = 0.98) was not significantly different to the second highest combination (MRMR and ADAC, AUC = 0.97) (*p*-value = 0.4795, DeLong test).

**Table 4:** The selected features with the best performance for LNM

Number	Sequence	Feature
1	ADC	Variance
2	ADC	S(1,1)Correlat
3	ADC	S(2,-2)SumVarnc
4	ADC	S(0,4)Entropy
5	ADC	S(5,0)SumAverg
6	ADC	Teta3
7	ADC	WavEnLH_s-4
8	DWI	Variance
9	DWI	S(2,0)DifEntrp
10	DWI	S(3,-3)SumOfSqs
11	DWI	S(4,0)Contrast
12	DWI	S(5,0)Entropy
13	DWI	45dgr_LngREmph
14	DWI	WavEnLL_s-4
15	T1WI	S(3,0)SumAverg
16	T1WI	S(3,3)SumVarnc
17	T1WI	S(0,5)SumEntrp
18	T1WI	S(5,-5)SumOfSqs
19	T1WI	S(5,-5)DifVarnc
20	T1WI	Vertl_RLNonUni
21	T1WI	WavEnLL_s-1
22	T1WI	WavEnHH_s-1
23	T2WI	Skewness
24	T2WI	S(0,1)DifVarnc
25	T2WI	S(2,0)SumAverg
26	T2WI	S(2,2)InvDfMom
27	T2WI	S(3,0)SumOfSqs
28	T2WI	S(5,-5)DifVarnc
29	T2WI	WavEnLH_s-2
30	T2WI	WavEnHH_s-4

Axial T1-precontrast weighted imaging, T1WI; axial T2-weighted imaging, T2WI; axial diffusion weighted imaging, DWI; Apparent diffusion coefficient, ADC

## Discussion

ECC is a malignant tumor with an extremely unfavorable prognosis despite the rare incidence of the disease. It’s important to comprehensively evaluate ECC, especially its DD and LNM, to guide clinicians and predict the prognosis of the tumor.

In this study, we found radiomics model incorporating ADC, DWI, T1WI, and T2WI had the highest diagnostic performance in discriminating high and medium-low DD groups of ECC (AUC = 0.90 (95% CI 0.75–1.00)), and LNM of ECC (AUC = 0.98 (95% CI 0.94–1.00)), suggesting that the clinical use of radiomics is promising in terms of the preoperative evaluation of ECC. Specifically, machine learning algorithm combinations of five feature selection methods and ten classification algorithms were applied to build radiomics signatures for DD and LNM of ECC. As a result, the algorithm combination of feature selection method JMI and machine learning classifier BAGC achieved the best predictive performances for DD with satisfying accuracy of ACC = 0.85 (95% CI 0.69–1.00) and AUC = 0.90 (95% CI 0.75–1.00) based on five selected optimal features. For LNM, the combination of feature selection method MRMR and classifier XGBC achieved the highest performance, with ACC = 0.90 (95% CI 0.77–1.00) and AUC = 0.98 (95% CI 0.94–1.00), based on 30 selected optimal features. The results demonstrated that radiomics analysis was able to accurately predict the DD and LNM for ECC cases. Meanwhile, the predictions achieved in radiomics analysis also had implications for guiding the clinicians in selecting the most appropriate treatment strategy and hopefully improving the prognosis of patients with ECC.

Recently, many studies have indicated that contrast-enhanced CT, PET-CT, and MRI played an important role in detecting of LNM of CCA [50–52]. It’s reported that PET-CT and MRI have been proposed to predict DD of CCA [53, 54]. However, discrimination of malignant from benign nodes and various DD on cross-sectional imaging with the traditional practice of visual interpretation remains challenging. These conventional imaging modalities based on morphologic criteria or metabolic activity still have some limits and are unable to fully meet the clinical requirements. In contrast, radiomics, which is more reflective of quantitative information drawn from images rather than those drawn by the naked eye, can enable mineable high-dimensional data to be applied within clinical decision support [55, 56]. The main contribution of this study is developing machine learning-based radiomics to predict LNM and DD of ECC using MRI data. For predicting LNM, the radiomics signature derived from ADC, DWI, T1WI, and T2WI sequences in this study achieved an AUC of 0.98, better than that derived from the evaluation of traditional images like PET-CT [9]. Besides, there is a lack of literature to identify the DD of ECC by using radiomics at present. Therefore, we developed a radiomics model to predict the DD of the tumor and achieved a better result with an AUC of 0.90.

At present, some radiomics methods have been used to evaluate CCA recently [26–30]. Wenjie Liang et al. constructed a novel nomogram for preoperative prediction of early recurrence (ER) in ICC, discovering the radiomics signature and clinical stages that could be used to predict ER of ICC after partial hepatectomy [26]. In another study, a combined model based on clinicoradiologic-pathological and radiomics features was developed to predict ER of ICC, with AUC, sensitivity, and specificity of 0.949, 0.875, and 0.774, respectively [27]. Besides, it’s reported that two radiomics models were built based on arterial phase (the highest AUC of 0.89) and portal venous CT scans (the highest AUC of 0.81) respectively to evaluate LNM and clinical outcome of biliary tract cancer in two previous studies, which were inferior to ours [28, 30]. Lei Xu and his partners used a radiomics approach based on MR images through a support vector machine for preoperative lymph node status evaluation in ICC, with AUC of 0.788 and 0.787 in the training and validation cohort, respectively, but still inferior to ours [29]. More importantly, almost no relevant studies were reported to evaluate DD and LNM of ECC simultaneously using radiomics developed by machine learning. In addition, as mentioned before, there exist many differences between ECC and ICC, e.g., origin, growth pattern, morbidity, imaging characteristics, and prognosis of tumor. Therefore, we separately built the radiomics models based on MRI of ECC in our study to predict DD and LNM of the tumor. This work contributes to the line of literature on MRI radiomics analysis of DD and LNM of ECC by developing a machine learning framework combining feature selections and classifications.

Of course, radiomics analysis is an analysis that relies on the quantity and quality of data. Especially for machine learning-based methods, the data size is one important determinant for the final predictive performance. The more data we used to train and test the algorithms, the more confidence we have in the obtained models and performance. Compared with other radiomics analyses, the sample size is limited in our study. This difficulty is not uncommon in radiomics studies, and small datasets still demonstrate the potentials of radiomics approaches [57–60]. Our study initially indicates that radiomics analysis has potentials in predicting DD and LNM of ECC with promising performance. The proposed methods could guide the treatment strategies and improve the quality of life in patients with ECC. In other words, radiomics analysis based on big data could help in cases of rare diseases. Therefore, the standardized data collection terminological systems and approaches such as ENT COBRA ONTOLOGY [61] and SKIN-COBRA [62] must be followed to ensure the high data quality and the high performance of machine learning algorithms.

Meanwhile, it’s worth mentioning that since we systematically invested 1000 combinations of feature selection methods and machine learning classifiers in this study, DeLong test [41] should be applied in pairwise comparing of two models. Only when *p*-value < 0.05 in DeLong test, the two given models could be considered significantly different. Therefore, we conducted DeLong test to all pairs, though the number of possible pairs was significantly larger than most previous studies in which only a few models were considered [42–49]. Similar to those studies in which the chosen models of optimal AUC values were not found significantly different to other models in DeLong test [43–45, 49], we could still choose and apply the reported model combinations (DD: JMI and BAGC; LNM: MRMR and XGBC) to achieve satisfying performance. However, they should not be treated as the only best models, since they were not significantly different to other combinations, especially not to other combinations of similar AUC values in DeLong test.

Our study still had limitations. Firstly, since machine learning-based approaches are data-driven, the development and the performance of machine learning algorithms rely on the quality and quantity of datasets. However, due to the rareness of ECC, the number of ECC cases used here was relatively small. Another limitation of the present dataset was that the unbalanced positive samples versus the negative samples due to the low incidence of ECC. Therefore, the initial results obtained in the present study should be treated with cautions. In the future, a larger-scale patient population will be acquired to further improve and validate the proposed machine learning-based radiomics models for DD and LNM predictions of ECC. A larger and relatively balanced dataset would not only allow more extra validation but also lead to more confident performance and more reliable predictive models. Secondly, our study was retrospective and from a single institution. Prospective multi-center studies with considerably large datasets are needed to further develop our radiomics prediction models to validate the effectiveness as well as generalization. Therefore, we suggest conducting multi-center clinical collaboration in the future to utilize a larger ECC dataset for further validations. Thirdly, the number of features was larger than the number of cases in the present study. Though, we conducted a feature selection process before performing the classifications. This effort partially alleviated the difficulty. However, larger datasets were still needed to further reliable validations. Therefore, more efforts were required to collect more data to further validate the reproducibility of the present results and thus improve the performance of the proposed machine learning framework. Finally, our texture extraction was based on two-dimensional analysis instead of three-dimensional delineation, which may contribute to the loss of texture information in the tumor. Therefore, a three-dimensional analysis of ECC could be carried out in future work.

## Conclusions

In conclusion, our MRI radiomics models based on optimal combinations of feature selection methods and machine learning classifiers demonstrate potentials in predictions for DD and LNM in ECC. Though the dataset used in this study is limited, future investigations using a larger dataset could further investigate the framework proposed in this study for better performance. This machine learning-based radiomics analysis provided a potential noninvasive method to evaluate ECC, which could guide the clinician to select the optimal treatment strategy depending on the individual situation and evaluate the survival prognosis in patients with ECC.

## Data Availability

The data used in this study is available from the corresponding author on reasonable request. The code used in this study is available at GitHub (https://github.com/gracewang723/EC-paper).

## Abbreviations

ECC: Extrahepatic cholangiocarcinoma
MRI: Magnetic resonance imaging
DD: Differentiation Degree
LNM: Lymph node metastasis
T1WI: T1 weighted imaging
T2WI: T2-weighted imaging
DWI: Diffusion weighted imaging
ADC: Apparent diffusion coefficient
AUPRC: Area under precision recall curve
ROC: Receiver operating characteristic
AUC: Area under the ROC curve
ACC: Accuracy
JMI: Joint mutual information
CCA: Cholangiocarcinoma
ICC: categorized as intrahepatic
US: Ultrasonography
CT: Computerized tomography
PET-CT: Positron emission computerized tomography
THRIVE: T1 high-resolution isotropic volume excitation
FOV: field of view
TR: Time of repetition
TE: Time of echo
NEX: number of excitations
MRCP: MR cholangiopancreatography
ROI: Regions of interest
MRMR: Minimum redundancy maximum relevance
SKB: Select K best using analysis of variance
SP: Select percentile
WLCX: Wilcoxon
*p*-value: Probability value
ADAC: Ada boosting classifier
BAGC: Bagging classifier
BNB: Bernoulli naïve Bayesian
DTC: Decision tree classifier
GNBC: Gaussian Naïve Bayesian Classifier
KNNC: K nearest neighborhood classifier
RFC: Random forest classifier
SGDC: Stochastic gradient descent classifier
SVMC: Support vector machine classifier
XGBC: eXtreme gradient boosting classifier
CI: Confidence interval

## Footnotes

1 available at http://www.eletel.p.lodz.pl/programy/mazda/
